# Absence of long-range diffusion of OmpA in *E. coli* is not caused by its peptidoglycan binding domain

**DOI:** 10.1186/1471-2180-13-66

**Published:** 2013-03-23

**Authors:** Gertjan S Verhoeven, Marileen Dogterom, Tanneke den Blaauwen

**Affiliations:** 1 Molecular Cytology, Swammerdam Institute for Life Sciences, University of Amsterdam, Science Park 904, Amsterdam, 1098 XH, The Netherlands; 2 FOM Institute AMOLF, Science Park 104, Amsterdam, 1098 XG, The Netherlands; 3 Bacterial Cell Biology, Swammerdam Institute for Life Sciences, University of Amsterdam, Sciencepark 904, Amsterdam, 1098 XH, The Netherlands

**Keywords:** Bacterial cell wall, Peptidoglycan, Outer membrane, Diffusion, FRAP, Cell membrane, OmpA

## Abstract

**Background:**

It is widely believed that integral outer membrane (OM) proteins in bacteria are able to diffuse laterally in the OM. However, stable, immobile proteins have been identified in the OM of *Escherichia coli*. In explaining the observations, a hypothesized interaction of the immobilized OM proteins with the underlying peptidoglycan (PG) cell wall played a prominent role.

**Results:**

OmpA is an abundant outer membrane protein in *E. coli* containing a PG-binding domain. We use FRAP to investigate whether OmpA is able to diffuse laterally over long-range (> ~100 nm) distances in the OM. First, we show that OmpA, containing a PG binding domain, does not exhibit long-range lateral diffusion in the OM. Then, to test whether PG interaction was required for this immobilization, we genetically removed the PG binding domain and repeated the FRAP experiment. To our surprise, this did not increase the mobility of the protein in the OM.

**Conclusions:**

OmpA exhibits an absence of long-range (> ~100 nm) diffusion in the OM that is not caused by its PG binding domain. Therefore, other mechanisms are needed to explain this observation, such as the presence of physical barriers in the OM, or strong interactions with other elements in the cell envelope.

## Background

Little information exists on the mobility of (integral) outer membrane proteins (OMPs) in the bacterial OM. Traditionally, the bacterial outer membrane is presented as a tight, gel-like barrier, with LPS packed together with cations in a crystalline matrix [1,2]. At the same time, experimental evidence suggests that integral outer membrane protein IcsA is able to diffuse laterally over micron-ranges in the OM [3]. Recent developments in live-cell protein labeling and (fluorescent) imaging technology are starting to elucidate the nature of protein dynamics in the bacterial OM.

For example, recent work on the mobility of integral OMP LamB suggests that it is confined to a region of size ~50 nm [4,5]. This was based on the motion of a marker bead or quantum dot attached to a surface-exposed biotinylated loop of LamB. The authors propose that the confinement is caused by LamB’s attachment to the peptidoglycan layer (PG) layer [6]. Furthermore, in pioneering experiments, proteins in the cell envelope of *E. coli* have been labeled using a reactive fluorescent dye [7,8]. It was found that the mobility of (at least some) cell envelope proteins was restrained at the cellular poles [7]. Also, it was found that the cell envelope contained both mobile and immobile proteins [7,8]. It should be noted that “immobile” in this context refers to the absence of long-range (> ~100 nm) diffusion, as only single-molecule techniques have sufficient spatial resolution to detect the complete absence of diffusion over short-range distances (below ~100 nm).

In [8] it was speculated that one of the major OM proteins of *E. coli,* OmpA, would be one of the “immobile” proteins in the OM due to its PG binding domain. The PG interaction of OmpA originates from a separate C-terminal domain in the bacterial periplasm, and genetically truncated OmpA-177 consisting of only the TM domain assembles into the outer membrane as efficiently as the full-length protein [9,10]. In this study, we have exploited these features of OmpA to determine its mobility *in vivo* using fluorescence recovery after photobleaching (FRAP), as well as to establish whether the presence of the PG binding domain has an effect on the mobility of the OmpA TM domain.

FRAP is a relatively simple technique to measure mobility and diffusion of fluorescent proteins inside living cells. For *E. coli*, it has been used to measure diffusion constants for GFP in the cytoplasm and periplasm [11,12], as well as for various GFP fusions to inner membrane proteins [12-14].

The full-length, processed OmpA protein (325 residues) consists of two domains, an N-terminal transmembrane (TM) domain of 170 residues, connected via a short 19-residue Ala-Pro rich hinge region to a C-terminal periplasmic domain of 136 residues [15]. The periplasmic domain plays a structural role by non-covalently tethering the OM to the peptidoglycan cell wall layer [16]. For a comprehensive review on OmpA structure and function see [17].

We have taken advantage from the availability of a red fluorescent protein reporter (mCherry, [18]) that fluoresces in the periplasm of *E. coli*[19-21] to create fluorescent OmpA variants with and without PG binding domain. We used the by-now standard approach of elongating the bacterial cells using the antibiotic cephalexin [8,11,12].

We find that full-length OmpA exhibits an absence of long-range (> 100 nm) diffusion in the OM. Surprisingly, removing the PG binding domain genetically does not increase protein mobility. From this we conclude that the absence of long-range diffusion of OmpA is not caused by its PG binding domain.

## Results and discussion

### Functionality of the constructs

In previous work, we have shown that full-length OmpA with a small C-terminal linker (LEDPPAEF), as well as truncated OmpA with an epitope tag (SA-1, [22]) inserted in the first surface-exposed loop, expressed from plasmid in the presence of wild-type OmpA, are properly assembled into the outer membrane [10].

In this work, we have constructed C-terminal mCherry fusions to the constructs mentioned above, creating OmpA-mCherry (full-length) and OmpA-177-(SA-1)-mCherry (truncated) (pGI10 and pGV30, respectively, see Table 1). Since its discovery as fluorescent periplasmic reporter in *E. coli*[19], mRFP1/mCherry [18] has been used in several fusion constructs that sub-localize to the PG/OM layer, without interfering with their function. For example, the OM lipoprotein Pal-mCherry [20] localizes to mid-cell and complements a *Pal* deletion, and PulD-mCherry [21] allows the formation of PulD multimers in the OM.

**Table 1:** Strains and plasmids

**Strains**	**Genotype**	**Reference**
LMC500 (MC4100 *lysA*)	F, *araD139*, Δ *(argF-lac)*U169, *deoC1*, *flbB5301*, *ptsF25*, *rbsR*, *relA1*, *rpslL150*, *lysA1*	[23]
DH5α	F, *endA1*, *hsdR17*(r_k_ m_k+_), *supE44*, *thi-1*, *recA1*, *gyrA*, *relA1*, Δ (*lacZYA-argF*)U169, *deoR*, Ф80 *lacZ*Δ M15	Lab collection
DH5α-Z1	DH5α LacI_q_^+^ TetR^+^	[24]
**Plasmids**	**Proteins expressed**	**Reference**
pGI10	pTHV037 OmpA-LEDPPAEF-mCherry	This work
pGV30	pTHV037 OmpA-177-(SA-1)-LEDPPAEF-mCherry	This work
pSAV47	pTHV037 mCherry-EFSR	[25]
pTHV037	pTRC99A with a weakened IPTG inducible promoter	[26]

Cells are grown in EZ defined rich medium [27] (see also Methods), with 0.2% glucose as carbon source. We refer to this medium as DRu (defined rich glucose) medium from now on. No adverse effects on growth rate were observed for either construct under the experimental growth and induction conditions reported here.

**Figure 1:**
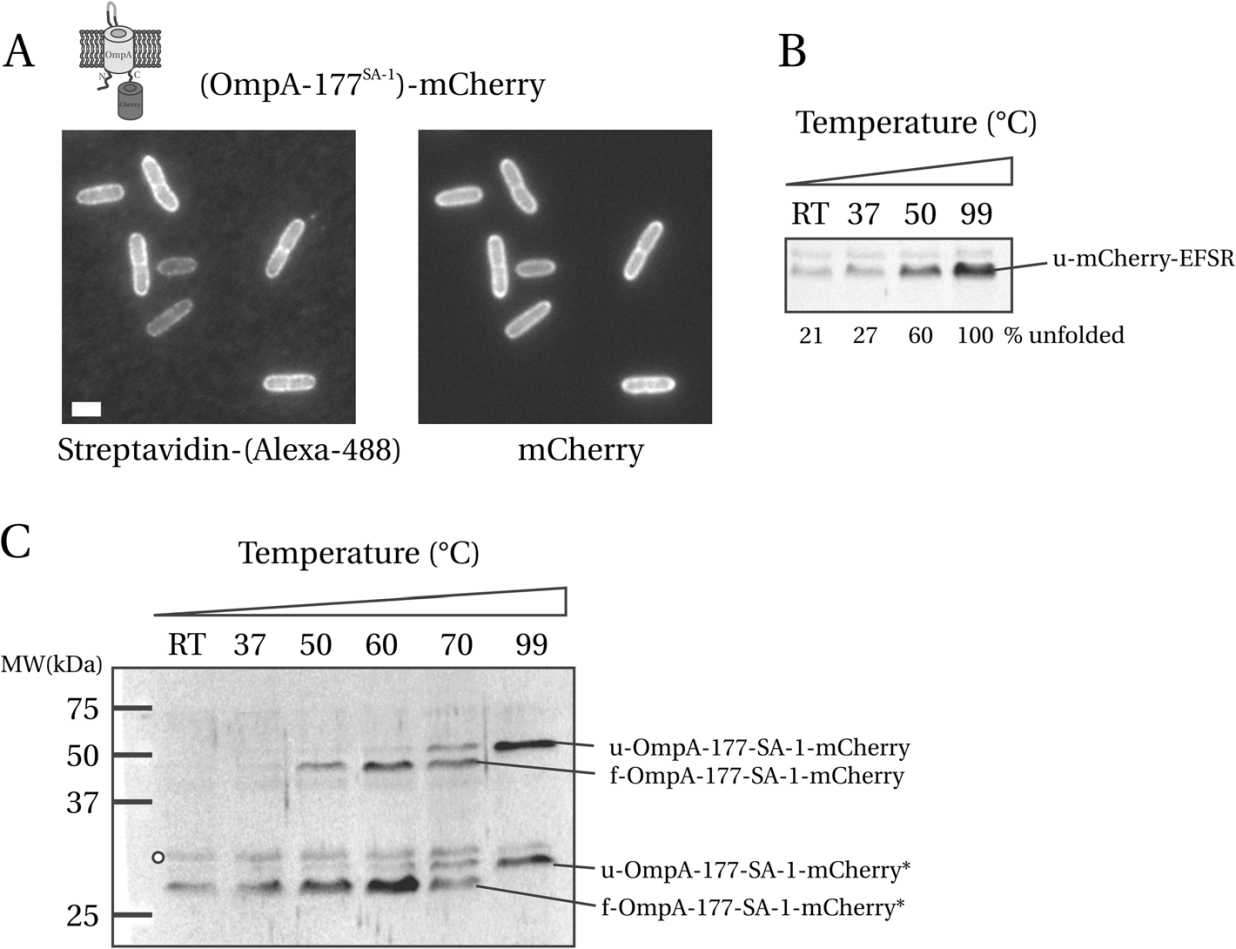
**OmpA-177-(SA-1)-mCherry is properly inserted in the OM. A**) Cells grown to exponential phase in DRu medium with 0.1 mM IPTG were labeled with fluorescent streptavidin. Scale bar is 1 × 2 μm. **B**) mCherry-EFSR is not heat-modifiable. Sonicated cell lysate of LMC500 expressing mCherry-EFSR was resuspended in sample buffer and either; not heated (RT), heated at 37°C for 5 min, heated at 50°C for 15 min, or heated at 99°C for 10 min. Shown is an immunoblot probed with anti-DsRed antibody. The faint band present in each lane is aspecific. The unfolded (denatured) mCherry-EFSR band is indicated. Percentage of unfolded mCherry-EFSR are indicated, assuming that after heating at 99°C all protein is unfolded. **C**) Heat-modifiability of OmpA-177-SA-1-mCherry. Cells from the same culture used for labeling in A) were sonicated and resuspended in sample buffer. Heat treatment as in B), heating at 60°C and 70°C was for 15 min. The folded and unfolded forms of both the intact fusion and the degradation product are indicated by a preceding f- or u-, respectively.

**Figure 2:**
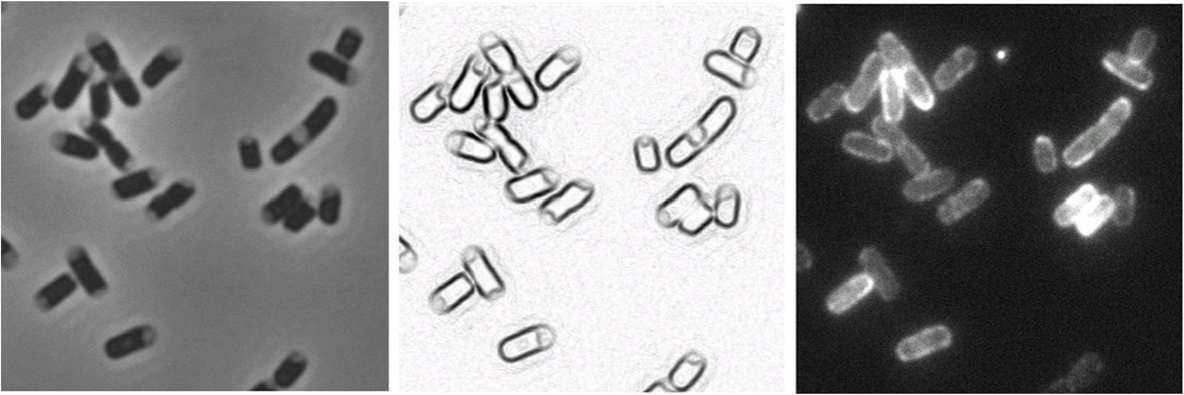
**OmpA-mCherry is associated with the PG/OM layer.** Cells expressing full-length OmpA-mCherry are plasmolyzed in hypertonic sucrose solution. Strain is LMC500. The fluorescence (right) remains associated with the PG/OM layer. Growth conditions: overnight TY culture, diluted 100x in fresh TY grown to exponential phase in 37°C. Plasmolysis is at RT. The phase-contrast image (left) is also depicted inverted-negative (middle) to more clearly visualize the plasmolysis bays.

LMC500 (MC4100 *LysA*) cells expressing either construct exhibit a red fluorescent halo along the cell’s perimeter (Figure 1A and Figure 2), as expected for fluorescence originating from the periplasm [28]. For cells grown to steady state, the fluorescence was distributed evenly along the cell perimeter, showing no preference for the cell pole, the cylindrical part or the division site. We tested if the truncate OmpA-177-(SA-1)-mCherry fusion was properly inserted in the OM using two different methods: (a) fluorescent imaging of live cells after staining the surface-exposed epitope tag, and (b) SDS-PAGE gel-shift experiments.

The cells are grown in the presence of IPTG to induce expression of the construct. After staining with fluorescent Streptavidin, we find that the SA-1 peptide is properly exposed on the cell surface (Figure 1A), suggesting that the OmpA TM domain is properly inserted in the OM, with the mCherry domain present in the periplasm.

We used SDS-PAGE gel-shift experiments to check if the constructs are intact or suffer from degradation, and if so to what extent. These experiments make use of OmpA’s so-called heat modifiability [29]: In its folded form, OmpA migrates to a different position in SDS-PAGE compared to its (heat denatured) unfolded form [9,10].

First, we checked for a possible heat-modifiability of mCherry, as it also has a β-barrel fold. To this end, we grew cells expressing cytoplasmic mCherry, lysed them by sonication, and after varying heat treatment, subjected the samples to SDS-PAGE followed by immunoblotting with a monoclonal antibody (anti-DsRed, Clontech) that recognizes only denatured DsRed variants, including mCherry (Figure 1B). Thus, we make use of the antibody’s specificity for the unfolded state of mCherry to obtain information on its folding state after varying heat treatment conditions. A band of the expected height (27 kDa) was present that increased in intensity upon heating (the faint band above it was also present in lysate without mCherry), and did not exhibit heat-modifiability. The increase in intensity is explained by a gradual unfolding of mCherry due to increasing exposure to heat. If we assume that after boiling, all mCherry is unfolded, we then conclude based on band intensities that at RT i.e. without any heat treatment, ~80% of mCherry is folded and 20% is not. Since mCherry unfolds partially under conditions where OmpA is fully stable (15 minutes at 50°C, [9]), we conclude that the mCherry β-barrel fold is less stable than that of the OmpA TM domain (Figure 1B). Therefore, the anti-DsRed can be used to determine the folding state of the OmpA TM domain, because the denatured mCherry will become visible before OmpA has unfolded, and any gel-shifts observed can be unequivocally attributed to the OmpA TM domain, because mCherry itself becomes visible only after it has unfolded, and does not exhibit heat modifiability.

To test the heat-modifiability of the OmpA-177-(SA-1)-mCherry fusion, an immunoblot containing cell lysates heated at different temperatures was probed with anti-DsRed (shown in Figure 1C). Starting at the far right lane (99°C), two bands are visible, a low and high molecular weight band (LMW and HMW respectively). At RT and 37°C, only a faint LMW (degradation) band at 26 kDa was detected. At 50°C, folded mCherry starts to unfold, and the fusion becomes visible on blot. From literature [9] and our own experiments, we know that the folded OmpA TM domain does not unfold at all at 50°C. Increasing the temperature further from 50°C to 99°C, the OmpA TM domain unfolds and the intact fusion (HMW band) shifts to its expected molecular weight of 49 kDa. These results demonstrate that the OmpA TM domain remains heat-modifiable and therefore is correctly assembled into the OM when mCherry is fused to its C-terminus.

With increasing exposure to heat, the initially faint LMW (degradation) band also increased in intensity, and displays the exact same heat-modifiability behavior as the intact fusion between the OmpA β-barrel and mCherry. Because we know that mCherry does not exhibit heat-modifiability, the degradation band must consist of the OmpA β-barrel with (based on a MW of 28 kDa and assuming C-terminal degradation) the N-terminal part of mCherry (~55 residues), which appears to contain the epitope recognized by the monoclonal antibody. We conclude that cells expressing OmpA-177-SA-1-mCherry contain a mixture of intact fusion assembled in the OM, and OmpA-177-SA-1 with a C-terminal part of mCherry proteolytically removed. Assuming C-terminal degradation, the removed part then contains the chromophore [30], and therefore this would represent a dark sub-population of OmpA TM domain in the OM.

For the full-length OmpA-mCherry fusion (pGI10), we already knew that the full-length OmpA with C -terminal linker, but without mCherry (pGI9), was inserted properly in the OM [10]. Therefore, we only checked that the mCherry fluorescence was associated with the PG/OM layer by fluorescence microscopy of plasmolyzed cells (Figure 2) [31]. This was indeed the case.

### FRAP results on cytoplasmic mCherry

To maximize the likelihood of observing OmpA mobility, we avoided the cell poles (poles contain inert PG and retain some OM proteins [7]) and performed the FRAP experiments in the cylindrical part of elongated cells. To create elongated cells (filaments) we grew the cells in the presence of the antibiotic cephalexin which blocks cell division but allows further elongation [11,12]. The effect of cephalexin on bacterial cells is well-known: it binds with high affinity to PBP3, interfering with its ability to function in cell division. In addition, it has recently been shown that PBP3 only interacts with PBP2 (part of the protein complex responsible for elongation) during division at mid-cell [32]. We expect therefore that the structure of the cell wall in filaments will be highly similar to that of normal length cells.

**Figure 3:**
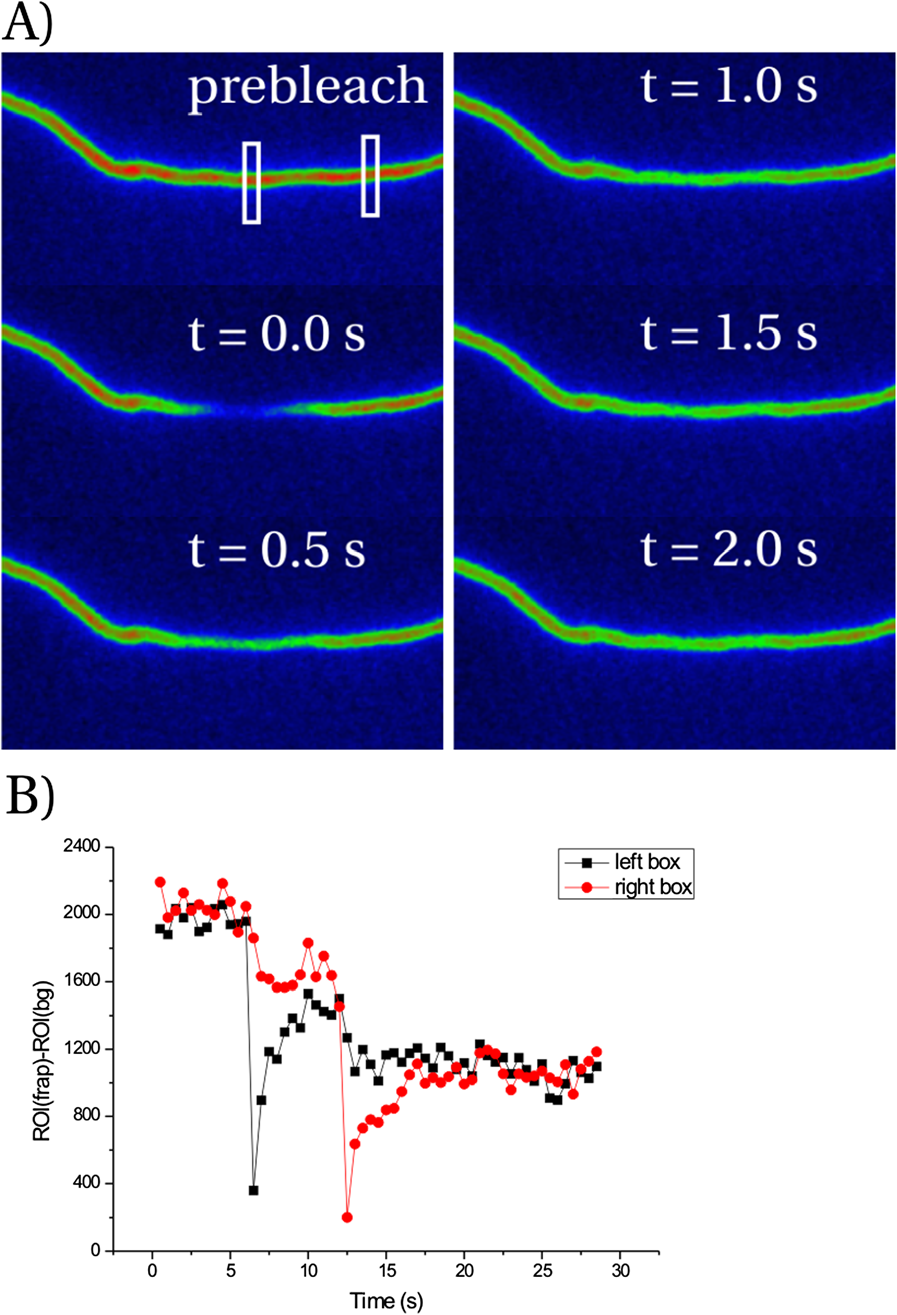
**Rapid recovery of cytoplasmic mCherry.** Filament imaged at 2 fps. Halftime of recovery is on the order of 1 s. A false color scale (ImageJ Rainbow RGB) is used to emphasize differences in intensity. A rectangular ROI box of 2 x 28 is positioned manually at the center of bleaching, and the average pixel intensity, corrected with the average background intensity is calculated. Two subsequent FRAP events are recorded, at two different locations. The two FRAP ROIs are drawn in the prebleach image. For the first FRAP pulse, the first few images are depicted in **A**). After each laser pulse, total fluorescence is also reduced by approx. 20% because during bleaching also the imaging continued at maximum laser power. This was corrected in subsequent experiments on OmpA (Figures 4 and 5). **B**) Pixel intensities after background subtraction for both the FRAP ROI (gray symbols) and a non-bleached reference ROI (red symbols) along the filament. Bacterial diameter is ~ 1 μm. Protocol: A fresh overnight culture of LMC500/pSAV047 grown in TY medium at 28°C is diluted 5000x into fresh TY medium and grown for 2 hours. Then cephalexin is added to induce filamentation and the cells are grown further for 2 hours. Next, the cells are concentrated 10x by centrifugation and resuspension. Then 2x 5 μl cells are added to a glass observation chamber containing TY agar with cephalexin and ampicillin (10 μg/ml and 100 μg/ml respectively). Finally, the cells are imaged in TIRF mode with epi-like TIRF angle.

We tested our setup by starting with cells expressing cytoplasmic mCherry, which should give a recovery rate similar to that observed for cytoplasmic GFP, for which diffusion constants of 6–9 μm^2^/s are reported [11,12]. The average length scale that corresponds with such a diffusion constant is $\sqrt{2\mathrm{Dt}}$ = 2–3 μm when t = 0.5 s. Indeed, the half-time to recover fluorescence in our bleached region of ~ 3–4 μm is on the order of 1 s (Figure 3), yielding a diffusion coefficient of 4–8 μm^2^/s.

### FRAP results on full-length OmpA-mCherry

As we were interested in diffusion / mobility of OmpA in the OM, and our timescale of observation is tens of minutes, we risked mistaking OmpA synthesis, OM insertion and / or fluorophore maturation for fluorescence recovery caused by lateral diffusion. To minimize this risk we adopted the following procedure: First the cells were grown to steady state in DRu medium in the presence of IPTG to induce expression (“pulse”), followed by resuspension of the cells in medium without IPTG to repress new synthesis (“chase”). Growing the cells in DRu medium for an additional 2 hours in the absence of IPTG allows time for export to finish and the mCherry fluorophore to mature. This way, we expected to end up with cells that contain little precursor or partially degraded protein. Then we transfered the filaments to the observation chamber (DRu-agar with ampicillin and cephalexin) and performed the FRAP experiment at room temperature. We made use of the Perfect Focus System that is part of the Nikon Eclipse Ti microscope system to keep the filament in focus during the experiment, which takes about 15–20 min per filament (N = 9).

**Figure 4:**
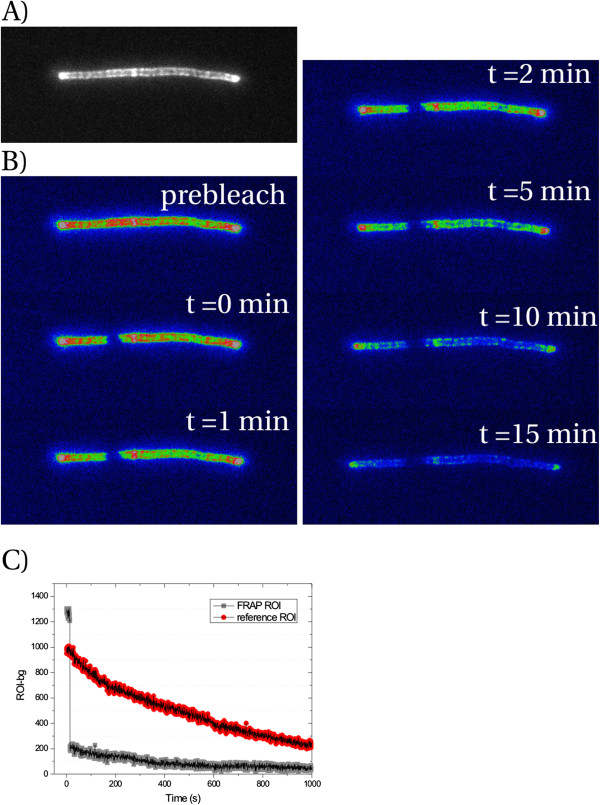
**OmpA-mCherry does not exhibit long-range lateral diffusion.** (**A**) Grayscale image. Note that the poles are brighter than the cylindrical part of the cell. (**B**) False color images. All images have the same color table (ImageJ Rainbow RGB) and are not contrast enhanced relative to each other. (**C**) Pixel intensities after background subtraction for both the FRAP ROI (gray symbols) and a non-bleached reference ROI (red symbols) along the filament. Acquisition rate was 2 fps.

In Figure 4 a representative image series is shown. Several observations can be noted. As is apparent, significant bleaching occurs (exposure time 100 ms, acquisition rate 2 frames per second (fps)). Furthermore, the filament is still growing in the chamber. This means that the OM can move with respect to the cover slip, and the cover slip should not interfere with the mobility (if any) of OmpA. Also, the poles are much brighter than the cylindrical part. This makes sense when OmpA-mCherry does not exhibit long-range lateral diffusion: because synthesis is shut down during elongation / filament formation, and cell wall growth occurs randomly along the cylindrical region, the existing OmpA-mCherry is diluted in the cylindrical part, but not in the poles, where no growth occurs [33]. Even after 15 min, no significant recovery had occurred. Thus, we conclude that full-length OmpA-mCherry is either immobile or its mobility is limited to distances below ~100 nm (our spatial resolution is limited by the pixel size). This was to be expected, since full-length OmpA is thought to be anchored to the PG layer underneath the OM.

### FRAP results on truncate OmpA-177-SA-1-mCherry

**Figure 5:**
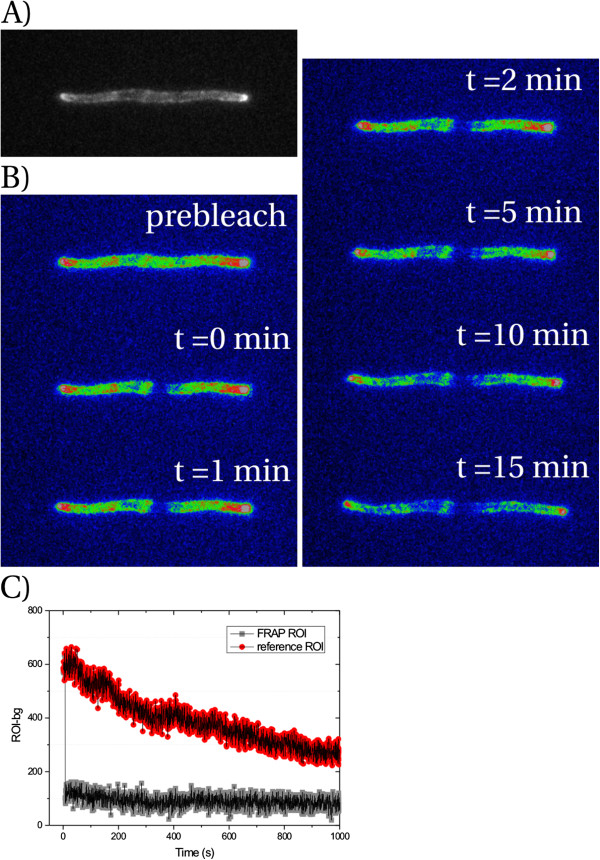
**OmpA-177-mCherry does not exhibit long-range lateral diffusion.** (**A**) Gray-scale image. (**B**) False color images. All images have the same color table and are not contrast enhanced relative to each other. (**C**) Pixel intensities after background subtraction for both the FRAP ROI (gray symbols) and a non-bleached reference ROI (red symbols) along the filament. Acquisition rate was 2 fps.

After genetic removal of the PG binding domain of OmpA, we expected that this would allow the fusion to laterally diffuse in the OM. To our surprise, the results obtained were essentially identical to those of full-length OmpA. All filaments observed (N = 7) did not show recovery on the timescale of 15 min. In Figure 5 a representative image series is shown. Again, we see that the poles are more fluorescent compared to the cylindrical part. Because we have observed on immunoblot that all OmpA-177 with (either intact or partially degraded) mCherry attached is heat-modifiable, we can conclude from these results that the OmpA-177-SA1-mCherry present in the OM is immobile or its mobility is limited to distances below ~100 nm.

## Conclusions

To conclude, we have observed that the OmpA-177 TM domain fused to mCherry, as well as full-length OmpA fused to mCherry, exhibit an absence of long-range (> ~100 nm) diffusion in the OM on a timescale of tens of minutes. Such absence of long-range lateral diffusion has been observed before, and PG interaction was invoked in explaining (part of) these observations [4,7,8]. Our results imply that other mechanisms are needed to explain our observations.

It is well established that OmpA is a monomer, in contrast to many other outer membrane proteins [34]. Immobilization through association with the endogenous OmpA proteins (that still contain a PG binding domain) can therefore not explain our observations. Possibly, an interaction with immobile LPS is responsible for the immobilization [8]. An alternative explanation could be the existence of sub-micron size domains in the OM acting as barriers to diffusion. Interestingly, recent *in vivo* single molecule fluorescence experiments performed for OMP’s OmpF and BtuB implied that OmpF diffused within domains of ~100 nm in the OM, and that on average, BtuB traversed 190 nm in 0.25 s, the longest time-scale for which results were reported [35].

It will be interesting to see whether the short-range diffusive properties of our constructs differ. This could be investigated using single-molecule techniques. Finally, we believe that our experimental design forms a valuable addition to existing techniques to study OM protein mobility, such as FRAP after chemical labeling treatments [8], tracking of single molecule fluorescence [35,36] as well as single particle tracking [4,5].

## Methods

### Strains and constructs

*E. coli* strains (Table 1) were grown at 37°C in TY medium containing 1% Bacto trypton, 0.5% Bacto yeast extract, 0.5% NaCl and 3 mM NaOH (for cloning and pre-cultures). For the FRAP experiments, strains were grown in defined rich medium with 0.2% glucose as the carbon source (Teknova M2105 Kit) and supplemented with 1 mM thiamine-HCl (Sigma). All constructs (Table 1) were cloned into a pTrc99A vector (Pharmacia Biotech, USA), a pBR322 derivative plasmid, of which the trc promoter was modified with a down mutation to reduce expression levels [26]. For induction conditions, cells were grown for an extended period (~15 hours) while keeping the OD550 below 0.2 in the continuous presence of 0.1 mM IPTG. Ampicillin (100 μg/ml) was used to maintain plasmids. LMC500 (MC4100 *lysA*) was made chemically competent using the calcium chloride method.

All DNA manipulation, analysis and bacterial transformations were performed according to standard protocols [37]. All PCR fragments were sequenced at the AMC DNA sequencing facility (Amsterdam Medical Centre).

pGV30 (proOmpA-177-SA1-LEDPPAEF-mCherry) was created as follows (Table 2 shows the primers used). An *XhoI* site was introduced at the C-terminus of OmpA-177 3xFLAG by PCR on pGV4 [10] using primers proOmpANcoIFW and OmpAXhoIPstIRV. This fragment was cloned into pTHV037 using *NcoI* and *PstI* sites, resulting in pGV14. The *Pal* gene excluding its signal sequence and the Cysteine that becomes acylated, was PCR-ed from the chromosome of LMC500 using primers PalXhoIFW and PalBamHIHindIIIRV. The PCR fragment was digested with *XhoI* and *HindIII* and ligated into *XhoI/HindIII* digested pGV14 to form pGV15 (proOmpA-177 L3 3xFLAG-Pal-LEDP). mCherry was PCR-ed from pSAV047 [25] using primers mCherryFW and mCherryHindIIIRV. This PCR fragment was digested with *BamHI* and *HindIII* and ligated into *BamHI/HindIII* digested pGV15 to form pGV16 (proOmpA-177 L3 FLAG-Pal-LEDPPAEF-mCherry). The LEDPPAEF linker was copied from [20]. OmpA-177 L3 FLAG was PCR-ed from pGV4 with primers proOmpANcoIFW and OmpAEcoRIRV, digested with *NcoI/EcoRI* and cloned into pTHV37 to form pGV17 (proOmpA-177 Loop 3 FLAG followed by 30 residues from the vector). A mCherry fragment from pGV16 was transferred to pGV17 via *EcoRI/HindIII* (proOmpA-177 L3 FLAG-mCherry) forming pGV18. OmpA-177-SA1 was PCR-ed from pB33OS1 [22] with primers proOmpANcoIFW and OmpAEcoRIRV, digested with *NcoI* and *EcoRI* and ligated into likewise digested pGV18 to form pGV30.

**Table 2:** DNA primers used in this study

**Name**	**Sequence**
proOmpANcoIFW	5-CGGCAGCCATGGCAAAAAAGACAGCTATCGCG-3
OmpAXhoIPstIRV	5-ATTACTGCAGTTAGCTCGAGGGAGCTGCTTCGCCCTG-3
PalXhoIFW	5-TTAACTCGAGCAACAAGAACGCCAGCAATGAC-3
PalBamHIHindIIIRV	5-TAGGAAGCTTAAGGATCCTCAAGGTAAACCAGTACCGCACGAC-3
mCherryFW	5-CCGGGATCCCCCCGCTGAATTCATGGTGAGCAAGGGCGAGG-3
mCherryHindIIIRV	5-TAATAAGCTTACTTGTACAGCTCGTCCATGC-3
OmpAEcoRIRV	5-ATTAGAATTCAGCGGGGGGATCCTCAAGTGGAGCTGCTTCGCCCTG-3

pGI10 was created as follows. A mCherry fragment from pGV16 was transferred to pGI9 (OmpA-LEDPPAEF) [10] via *EcoRI/HindIII*. All cloning was performed in either DH5α-Z1 or DH5α (Table 1).

### FRAP experiment

Cells are grown for ~15 hours to exponential phase in EZ defined Rich glucose (DRu) medium with 100 μM IPTG at 28°C (“pulse”). Then at OD550 < 0.2, cells are washed two times with DRu medium, and diluted to OD~0.05. Cephalexin and ampicillin are added at a concentration of 10 and 100 μg/ml respectively and the cells are grown for an additional 2 hours (“chase”). Then, the filaments are incubated for 30 min at room temperature. Imaging is at room temperature. The sample consists of two object slides, one of which has an oval shape mechanically cut out, stuck together using vacuum grease (see also [38]). Molten DRu agar containing cephalexin and ampicillin is poured inside, and a silanized cover slip is added to create a flat agar surface. After the agar has solidified, the silanized slip is removed, the agar is allowed to dry in for 5 min, before 2 × 5 μl cells are pipetted on the agar. Finally, a chromo-sulfuric acid cleaned cover slip is placed on top and fixed in place with vacuum grease. This creates a sealed chamber with the elongated cells lying on the agar, and the imaging is through the cover slip. The setup consists of a Nikon Eclipse Ti inverted TIRF/epi microscope equipped with a MAG Biosystems FRAP-3D unit and a Photometrics QuantEM 512SC EM-CCD camera (Roper Scientific), controlled with Metamorph software. A laser system provides green light at 561 nm. Typical FRAP setting is 100% power, duration 5–50 ms. Imaging mode is TIRF in epi-mode (TIRF angle ~90°), Nikon’s Perfect Focusing System (PFS) is used to keep filament in focus during the time-lapse imaging after bleaching. We verified with z-stacks containing slices of ~100 nm that both sides of the bacterium were bleached and imaged. Acquisition rate was every 0.5-10 s, depending on the experiment. Exposure times are typically 100–300 ms.

### FRAP analysis

The raw image TIFF stack (16 bit) is cropped, and (if necessary) registered using the ImageJ plug-in Stackreg (Rigid body setting), and rotated such that the filament long axis is aligned with the square ROI. Then, a square ROI of 4 × 44 pixels (1,5x lens) is used to quantify background signal (in a region without cell), a reference signal (a part of the filament that is not bleached) and the FRAP signal, the location where the fluorescence is bleached away. The average pixel intensity of the ROI is used. The ImageJ Multi-measure plug-in is used to measure all three ROIs for a single stack. The background is subtracted from both the reference and FRAP ROI. For the analysis of images taken with the 1x lens (Figure 3) a smaller region of 2 × 28 was chosen. The pixel size was ~100 nm.

### Cell fractionation, SDS-PAGE and immunoblots

For preparation of cell lysates, fractionation of cell lysates and immunoblots, see also [10]. For SDS-PAGE, samples were mixed with sample buffer (end concentration: 62.5 mM Tris pH 6.8, 2% SDS, 10% glycerol, 2% 2-mercaptoethanol), received heat treatment varying from incubation at RT to heating to 99°C for 5 min, and were finally electrophoresed on 15% polyacrylamide slabs. The bio-rad semi-dry blotting apparatus was used for immunoblotting. The anti-dsRed monoclonal antibody (#632392, Living colors series) was purchased from Clontech. The bands were detected using the ECL+ chemiluminescence kit (Amersham) and scanning with the STORM 860 fluorescence imager.

### Plasmolysis protocol

The protocol was taken from [31]. Overnight cultures of LMC500 cells expressing pGI10 were diluted 100x and grown for ~3 hours to an OD600 of ~0.5. Cells were grown in the absence of the inducer. 2x 500 μl cells were transferred to eppies. To prepare cells for fluorescence microscopy, 0.5 ml of culture was pelleted and resuspended in 10 μl of Luria-Bertani medium (control) or 10 μl of plasmolysis solution (15% sucrose, 25 mM HEPES [pH 7.4], 20 mM NaN_3_). One microliter of control cells or plasmolyzed cells was immobilized on a thin layer of 1% TY agarose or of 1% agarose in 15% sucrose in HEPES (to maintain plasmolysis), respectively. Live cells were visualized by epifluorescence microscopy within 15 min of slide preparation with a Olympus BX microscope equipped with a Coolsnap FX charge-coupled device camera.

## Competing interests

The authors declare that they have no competing interests.

## Authors’ contributions

All authors conceived the study, designed the experiments and participated in data analysis and interpretation. GSV carried out the experiments and drafted the manuscript. All authors read and approved the final manuscript.
